# Calcium Modulation of Toxicities Due to Cisplatin

**DOI:** 10.1155/MBD.1998.77

**Published:** 1998

**Authors:** Surinder K. Aggarwal

**Affiliations:** Department of Zoology Michigan State University East Lansing Michigan 48823-1115 USA

## Abstract

Cisplatin (CDDP) is a potent anti-neoplastic agent with associated toxicities, especially gastrointestinal and nephrotoxicity that are its dose-limiting factors in clinical oncology. In an attempt to elucidate its mechanism(s) of action, liver and kidney tissues from normal and CDDP treated (1.8 mg/kg) dogs were evaluated for changes in various dehydrogenases [MDH, SDH, β-HBDH, IDH and G-6-PDH] and nonspecific lipase enzymes. CDDP treatment induced an inhibition of all the enzymes studied except G-6-PDH and nonspecific lipases, where there was a significant increase. Supplemental pretreatments with calcium 2.50 mg (150,000 USP units) ergocalciferol plus 1000 mg of elemental calcium as Tums 500 (EffeCal; calcium carbonate)/day seemed to retain enzyme levels close to normal with no apparent toxic side effects observed after CDDP. Calcium supplements post-CDDP treatment did not have any protective effect.


### CALCIUM MODULATION OF TOXICITIES DUE TO CISPLATIN

Surinder K. Aggarwal

Department of Zoology, Michigan State University, East Lansing, Michigan 48823-1115, USA

**Abstract**

Cisplatin (CDDP) is a potent anti-neoplastic agent with associated toxicities, especially gastrointestinal and nephrotoxicity that are its dose-limiting factors in clinical oncology. In an attempt to elucidate its mechanism(s) of action, liver and kidney tissues from normal and CDDP treated (1.8 mg/ kg) dogs were evaluated for changes in various dehydrogenases [MDH, SDH, β-HBDH, IDH and G-6-PDH] and nonspecific lipase enzymes. CDDP treatment induced an inhibition of all the enzymes studied except G-6-PDH and nonspecific lipases, where there was a significant increase. Supplemental pretreatments with calcium 2.50 mg (150,000 USP units) ergocalciferol plus 1000 mg of elemental calcium as Tums 500 (EffeCal; calcium carbonate) /day seemed to retain enzyme levels close to normal with no apparent toxic side effects observed after CDDP. Calcium supplements post-CDDP treatment did not have any protective effect.

### INTRODUCTION

Cisplatin (*cis*-dichlorodiammineplatinum II; CDDP), a broad spectrum anticancer agent has severe toxic side effects including nephrotoxicity, gastrointestinal toxicity and hypocalcemia. Nephrotoxicity manifests pathologically, as renal tubular damage which results in elevation of the blood urea nitrogen and serum creatinine levels.[1,2] Hydrating patients markedly diminishes kidney toxicity. Antioxidants and thiol containing compounds, such as sodium thiosulfate, have become part of the treatment regimen in an attempt to alleviate nephrotoxicity.[3] Despite these advances, nephrotoxicity is still a major concern. Similarly, various antiemetics are being used to alleviate emesis at great costs.[4] We can treat the symptoms but still are unable to point out the cause(s) of these toxicities. Although newer compounds are being synthesized with substitutions of various ligands to increase its efficacy and decrease its toxicities. In the process we may learn more about the structural and functional relationship.

Gastrointestinal toxicity in rats is manifest as bloating of the stomach and severe diarrhea as the rats do not have vomiting reflexes. Dogs however, do show vomiting reflexes and diarrhea in response to CDDP treatments. Calcium supplements have been shown to prevent stomach bloating in rats, protect enzyme function and preserve overall organ function by minimizing the disruption of cellular homeostasis initiated by CDDP treatment.[5-7] Present study was undertaken to characterize CDDP-induced changes in the various dehydrogenases involved in the glycolysis process and Kreb's cycle responsible for ATP production using dog kidney and liver tissues. Effort was made to explore the protective effects of calcium supplements on the dehydrogenases to prevent severe toxicities associated with CDDP treatment.

### MATERIALS AND METHODS

*Animals and Treatment*:

Male dogs weighing 70-95 lbs were kept on a 12 h light/12 h dark cycle. The dogs had free access to water and food. Animals (2) were administered CDDP (1.8 mg/kg) in 500 ml of 0.85% NaCl as an infusion over a period of 3 hours. Another group of (2) normal dogs were given orally 2.50 mg (500,000 USP units) of ergocalciferol (Banner Pharmacaps Inc., Elizabeth, NJ 07207) and 1000 mg of elemental calcium as Tums 500 (EffeCal; calcium carbonate) (Smithkline Beechum, Pittsburgh, PA 15230) daily for 15 days before treating them with CDDP and every day after CDDP infusion to maintain an elevated level of serum calcium. CDDP treated dogs were given ergocalciferol plus Tums 500 as a post-treatment. Blood and urine samples were collected at 12 h intervals for 3 days after CDDP treatments and once daily there after.

*Blood and Urine Analysis*:

Blood and urine samples were analyzed for ionic calcium levels using 634 calcium / pH analyzer. Blood urea nitrogen (BUN) and creatinine levels were monitored in accordance with the established methods.[8]

*Tissue Collection*:

The dogs were given a lethal dose of sodium pentobarbital (325 mg/kg). The kidney and liver tissues were quickly excised and mounted on cryostubs in O.C.T. medium (Miles Laboratories) and frozen at -20°C. Sections (10 µm) were cut for enzymatic analysis.

*Enzyme Localization*:

Frozen sections (10 µm) of normal, and treated animal tissues were picked up on standard glass coverslips and allowed to dry at room temperature. Succinate dehydrogenase (SDH - EC 1.3.99.1), glutamate dehydrogenase (GDH - EC 1.4.1.2), β-hydroxybutyrate dehydrogenase (HBDH - EC 1.1.1.30), malate dehydrogenase (MDH - EC 1.1.1.37), isocitrate dehydrogenase (IDH - EC 1.1.1.41), and glucose-6-phosphate dehydrogenase (G6-PDH - EC 1.1.1.49) were then localized, histochemically, by the standard Nitro BT method.[9] Sections were incubated at 37° C for 20 min with preheated media. The substrate or the coenzymes were omitted from the incubation media to serve as controls. Nonspecific lipase (EC 3.1.1.3) activity was localized by incubation of tissue sections in a medium according to the standard methods.[10,11] All slides were viewed with a Zeiss photomicroscope II and micrographs of random sections and random areas were prepared for quantitative analysis.

*Quantitative analysis*:

Staining intensity was based on an arbitrary scale from very intense response (+++++) to intense response (++++) to moderate response (+++) to poor response (++) to very poor response (+) to no response (-). To avoid any variations in the staining intensities due to section thickness approximately five transmission images from five sections each of the normal, CDDP, CDDP plus ergocalciferol/calcium treated and ergocalciferol/calcium plus CDDP treated tissues, were examined by the Zeiss 10 Laser Scanning Confocal Microscope (LSM). Quantitative analyses were made using the 'histogram' computer program. Random areas of the tissues were analyzed for staining intensity by the computer and a representative histogram detailing 'gray scale values' was produced for each enzyme. Statistical analysis was performed and gray scale values were then converted to percentages based on normal staining being equivalent to 100% intensity.[12]

### RESULTS

*Effects of Various Treatments on Dehydrogenases*:

Histochemically, of all the dehydrogenases studied (MDH, SDH, GDH, β-HBDH, IDH and G-6-PDH), only G-6-PDH demonstrated an increase after CDDP, treatment (see Table I). Thus, for the sake of simplicity, only MDH, and G-6-PDH will be discussed in detail here.

Sections of normal kidney and liver tissues, incubated for MDH localization demonstrated a dark blue granulation with intense diffuse staining throughout the cytoplasm of the cells. In the normal kidney, staining intensity and localization was approximately the same in both the cortical and medullar regions with the proximal and distal tubules having equally pronounced enzyme localization. For the sake of uniformity most of the observations described here are restricted to the pericentral regions of the liver.

In CDDP treated tissues, MDH staining was significantly decreased compared to normal. However, in ergocalciferol plus Tums 500 pretreated animal tissues, MDH staining and localization were very similar to that of the normal tissues. Post-treatment of the CDDP treated animals with ergocalciferol and Tums 500 did not show any protective effects of calcium (Table I).

Sections of normal tissues stained for G-6-PDH localization demonstrated a diffuse staining throughout the cytoplasm. However, after CDDP treatments, G-6-PDH staining was much more enhanced compared to the normal tissues with the exception of ergocalciferol/calcium plus CDDP treatment where the G-6-PDH levels were similar to the normal tissues. Again, ergocalciferol and Tums 500 administration after CDDP treatment did not show any deviations from that of the CDDP treatment alone (Table I).

*Effects of Various Treatments on Non-specific Lipase Activity:*

Under the light microscope non-specific lipase activity was observed as brown diffuse granulation throughout the tissues . In the kidneys, staining was similar in the proximal and distal tubules of the cortex and medulla. CDDP treatment caused an elevation in lipase throughout the kidney tubules and the hepatocytes as compared to the normal. After calcium pretreatment followed by CDDP the level of lipase was close to that of the normal tissues.

Table I. Average enzyme histochemical staining intensity for normal,and CDDP- treated tissues.[a]

| Treatment | SDH | GDH | HBDH | MDH | IDH | G6PDH | Lipase |
|---|---|---|---|---|---|---|---|
| 1. Normal | ++++ | ++++ | ++++ | ++++ | ++++ | +++ | +++ |
| 2. CDDP | + | + | + | + | + | +++++ | +++++ |
| 3. Ergocalciferol plus CDDP | +++ | +++ | +++ | +++ | +++ | +++ | +++ |
| 4. CDDP plus ergocalciferol | + | + | + | + | + | +++++ | +++++ |

[a] = +++++, very intense reaction; ++++, intense reaction; +++, moderate reaction; +, very poor reaction.

*Dog Serum Concentrations of $[Ca^{2+}]_i$ after Ergocalciferol:*

In order to maintain a constantly higher than normal levels of serum calcium, ergocalciferol and calcium carbonate in the diet proved to be very effective . Higher than normal level (1.5 m mol / L) of serum calcium were achieved by day 7 through ergocalciferol and Tums500 administration. Subsequent cisplatin infusions did not decrease the ionized serum calcium levels below normal (1.25-1.45 m mol/L). After 3 such cisplatin treatments extending over 9 weeks the blood urea nitrogen (BUN), and creatinine levels were found to be normal.

### DISCUSSION

CDDP, under low intracellular chloride ion concentrations, has been shown to hydrolyze into variously charged reactive species including monoaqua $[cis\text{-}(NH_3)_2PtCl(H_2O)]^+$ and diaqua-equated $[cis\text{-}(NH_3)_2Pt(H_2O)_2]^{2+}$ forms.[13-16] It is these hydrolyzed forms of CDDP (diol) that have been shown to be 1000 times more reactive than CDDP, and act through the inhibition of mitochondrial respiration by inducing uncoupling of oxidative phosphorylation.[16] This results in an efflux of calcium from the mitochondria and a temporary increase in the cellular calcium levels, which is thought to play a significant role in the disruption of normal calcium homeostasis, and hence cell function. In vitro studies have demonstrated that the energy requiring calcium transport in the mitochondria can be measured by the increased oxygen consumption after diol . This oxygen consumption is directly proportional to the concentration of diol used and can be reversed by SH rich N-acetyl-L-cysteine (NAC).[17] CDDP in an unhydrolyzed state does not seem to have any effect.

Mitochondrial glutathione (GSH) seems to be essential in the regulation of inner mitochondrial permeability and enzyme function by keeping SH in the reduced state.[18] When the SH-groups of enzymes are not maintained in a reduced form, they become inactivated.[19] CDDP-induced toxicities, especially nephrotoxicity, seem to be related to a decrease in the intracellular concentrations of GSH and protein bound SH-groups.[20,21]

NADH, which helps to maintain SH groups, declines with CDDP treatment. Consequently, this depletion of GSH and NADH appears to result in the inhibition of some dehydrogenases, resulting in the uncoupling of oxidative phosphorylation leading to hydroxyl radical formation and oxidative stress.[17,22] These free radicals attack polyunsaturated lipids and proteins and initiate lipid peroxidation.[23-25] This process becomes autolytic and causes severe damage to membrane integrity.[26]

Hypermetabolism is a cellular means of compensation for increased energy needs as a result of mitochondrial damage but this also leads to the additional activation of unregulated $Ca^{2+}$-dependent degradative enzymes such as phospholipases.[26] Phospholipases A & C are known to be activated after CDDP treatment.[27,28] Our studies have demonstrated such increases but these can be reversed by calcium supplements only prior to CDDP treatment. Similar to lipase activity G-6-PDH is also enhanced after CDDP treatment. It seems that elevation of G-6-PDH activity occurs because of its unique and critical function in NADH generation and maintenance of reduced sulphydryl groups as part of the pentose phosphate pathway. Possible lack of essential sulphydryl groups may be limiting its own inhibition allowing for increased activity. This may be a means for cells to adapt to the effects of CDDP

*In vitro* and *in vivo* studies have suggested calcium to modulate CDDP-induced toxicities.[17] In treatments with CDDP the importance of maintaining normal or close to normal levels of calcium is very suggestive of a competitive binding between $[Ca^{2+}]_i$ and $cis[(NH_3)_2Pt(H_2O)_2]^{2+}$ and the cellular membranes. *In vitro* experiments using MeroCalmodulin-1, a calcium-sensitive fluorescent analog of calmodulin it has been demonstrated that only the hydrolyzed form of CDDP is able to inhibit the conformational change which occurs after calcium binding to the molecule.[29] This conformational change which is calcium activated makes the molecule fluoresce.[29] Further, competitive binding studies for the calcium binding sites of the calmodulin molecule have demonstrated that it is only the diol form of CDDP that is able to inhibit the conformational change.[7] Similarly, acetylcholine release inhibition has been tied to the hydrolyzed form of CDDP in the rat stomach smooth muscle resulting in its bloating and ulceration.[6] However, these adverse effects of CDDP can be inhibited by calcium supplements only when calcium is administered in advance of CDDP. If CDDP is administered first and calcium is administered later then the various toxicities especially nephrotoxicity due to CDDP are not prevented. Probably once the $[Ca^{2+}]_i$ binding sites are blocked by diol, then the normal functions are disrupted especially various transport functions across the membranes through the inhibition of ATP.[5]

In conclusion, CDDP's disruption of calcium homeostasis initiates primary events such as lipid peroxidation and enzyme inhibition. These events damage the cells through mitochondrial damage, inhibition of mitochondrial function, depletion of ATP and other cofactors. This probably leads to apoptosis and tissue necrosis.[30] Thus, it seems that elevated calcium levels, via calcium supplementation, may act as another means of cytoprotection, by competing for binding sites with diol and prevent various toxicities associated with it.

### ACKNOWLEDGMENTS

Our thanks to Dr. R. Walshaw for his help with the dog studies and Dr. R. Nachreiner for his help with the ionized calcium determinations. Our special thanks to Andrulis Pharmaceutical Corporation, Bristol Meyers and NIH for the samples of cisplatin. Supported by All-University Research Initiation Grants.

### REFERENCES

1. Rosenberg B, Van Camp L, Krigas T. *Nature* 1965; **205**: 698.
2. Hardaker WT, Stone RA, McCoy R. *Cancer* 1974; **34**: 1030.
3. Powis G, Hacker MP. *The toxicity of anticancer drugs*, New York, Pergamon Press. 1991
4. Moore AS, Rand WM, Berg G, L'Heureux DA and Dennis RA. J Am Vet Assoc. 1994; **205**: 441.
5. Aggarwal SK, Fadool JM. *Anti-Cancer Drugs* 1993; **4**: 149.
6. Aggarwal SK, San Antonio JD, Sokhansanj A, Miller C. *Anti-Cancer Drugs* 1994; **5**: 177.
7. Jarve RK, Aggarwal SK. *Cancer Chemother Pharmacol* 12997; **39**: 341.
8. Lelieveld P, Van der Vijgh WJF, Van Velzen D. *Eur. J.Cancer Clin. Oncol.* 1987; **23**: 1147.
9. Van Noorden CJF Butcher RW. *J Histochem Cytochem* 1984; **32**: 998.
10. George JC, Ambadkar RM. *J Histochem Cytochem* 1963; **11**: 420.
11. George JC, Iype PT. *Stain Technol* 1960; **35**: 151.
12. Jonker A, Geerts WJC, Charles R, Lamers WH and Van Noorden CJF. *J. Histochem Cytochem* 1995; **43**: 1027.
13. Jennerwein M, Andrews PA. *Drug Metabol Disp* 1995; **23**: 178.
14. Beaty JA, Jones MM, Ma L. *Chem Res Toxicol* 1992; **5**: 647.
15. Tinker ND, Sharma HL, McAuliffe CA. Qualitative investigation, both in vivo and in vitro, of the metabolites formed by cisplatin and paraplatin inbolbing high performance liquid chromatography analysis. In: Nicolini M, ed. *Platinum and other metal coordination compounds in chemotherapy*, Boston, Martinus Nijhoff, 1987.pp144-159.
16.. Aggarwal SK, Broomhead JA, Fairlie DP, Whitehouse MW. *Cancer Chemother Pharmacol* 1980; **4**: 249.
17. Aggarwal SK. *J Histochem Cytochem* 1993; **41**: 1053.

18. Reed DJ. Mechanisms of chemically induced cell injury and cellular protection mechanismn. In: Hodgson E, Levi PE eds. *Introduction to Biochemical Toxicology.* Appleton and Lange, Norwalk, CT. 1994; pp 264-295
19. Bogin E, Marom M, Levi Y. *Eur J Clin Chem Clin Biochem* 1994; **32**:843.
20. Levi J, Jacobs C, Kalman S, McTighe M, Weinder MW. *J. Pharmacol. Exp. Ther.* 1980; **213**: 545.
21. Batzer MA, Aggarwal SK. *Cancer Chemother Pharmacol* 1986; **17**: 209.
22. Zhang JG, Lindup WE. *Biochem Pharmacol* 1993; **45**: 2215.
23. Bompart G. *Toxicol Lett* 1989; **48**: 193.
24. Bompart G, Orfila C, Manuel Y. *Nephron* 1991; **58**: 68.
25. Vermeulen NP, Baldew GS. *Biochem Pharmacol* 1992; **44**: 1193.
26. Tyler D. *The mitochondrion in health and disease*, New York, VCH Publishers, Inc. 1992.
27. Van Rensburg CE, Van Staden AM, Anderson R. *Cancer Res* 1993; **53**: 318.
28. Nishio K, Sugimoto Y, Fujiwara Y, Ohmori T, Morikage T, Takeda Y, Ohata M, Saijo M. *J Clin . Invest* 1992; **89**: 1622.
29. Hahn KH, Waggoner AS, Taylor DL. *J Biol Chem* 1990; **265**: 203 .
30. Timbrell JA. *Principles of Biochemical Toxicology*, 2nd ed. London, Taylor & Francis. 1991

**Received: January 12, 1998 - Accepted: February 2, 1998 - Received in revised camera-ready format: February 2, 1998**

